# Cleansing Mechanisms and Efficacy on Artificial Skin

**DOI:** 10.3390/molecules30081813

**Published:** 2025-04-17

**Authors:** Tatiana Slavova, Rumyana Stanimirova, Krastanka Marinova, Krassimir Danov

**Affiliations:** 1 Department of Chemical & Pharmaceutical Engineering, Faculty of Chemistry & Pharmacy, Sofia University “St. Kliment Ohridski”, 1164 Sofia, Bulgaria; tatiana_slavova@lcpe.uni-sofia.bg (T.S.); rs@lcpe.uni-sofia.bg (R.S.);; 2 Centre of Competence “Sustainable Utilization of Bio-Resources and Waste of Medicinal and Aromatic Plants for Innovative Bioactive Products” (BIORESOURCES BG), 1164 Sofia, Bulgaria; 3 Centre of Competence “Smart Mechatronics, Eco- and Energy Saving Systems and Technologies”, 1164 Sofia, Bulgaria

**Keywords:** skin cleansing mechanisms, roll-up, artificial skin, surfactant interfacial activity

## Abstract

A systematic study on the mechanisms of cleansing artificial skin by solutions of widely used in personal care surfactants disodium laureth sulfosuccinate (DSLSS), sodium laureth sulfate (SLES), sodium dodecyl sulfate (SDS), dodecyl trimethyl ammonium bromide (DTAB), and coco glucoside (CG), is presented. The systematic characterization of soil removal from artificial skin revealed two primary cleansing mechanisms: emulsification and roll-up. Emulsification occurs in systems with very low interfacial tension, such as sebum in SLES solutions, while dimethicone soil was only removed by roll-up. The roll-up effectiveness depends on the surfactant’s interfacial activity and its adsorption on the soiled surface. Thus, the strong adsorption of DTAB on the skin leads to dimethicone roll-up at a relatively high interfacial tension of 11 mN/m. The anionic and nonionic surfactants adsorbed less at the artificial skin surface, and the oil/water interfacial tension value lowering below 5 mN/m is necessary for the roll-up to occur. Nonionic CG removed dimethicone at a lower concentration than ionic surfactants. Combining CG with ionic surfactants improved cleaning at lower total concentrations. Surfactant mixtures are used to formulate simple cleansing formulations, whose performance is also investigated by the developed in vitro approach. The results obtained allow for a good rating of the formulations, which correlates well with the performance of the surfactant mixtures and their interfacial activity.

## 1. Introduction

Nowadays, cleansing the skin is an innate concern for healthy skin with a good esthetic appearance. It provides removal of dirt, sebum, dead skin cells, cosmetic ingredients, e.g., dimethicone, pigments, etc. Cleansing and skin care play an important role in human psychological well-being and social interaction [1,2,3]. In the last decade, the cosmetic industry has provided myriads of products for skin cleansing based on various types and origins of the used surfactants, enormously enriched with “green” and “bio” sources, and their mixtures [4,5]. The efficacy and safety are the mandatory requirements for the surfactants and the raw materials included in the final products [1,3,4,6].

The human skin surface has been the subject of numerous studies in relation to the wetting of skin by water [7], surfactant mildness and skin irritation [1,4,8,9,10], and the surfactants’ effect on the wetting and lipid composition [11,12,13]. The basic understanding of any possible interactions and overall impact stems from the proper determination and interpretation of the skin surface energy [8,11,12,13,14,15,16,17]. In modern times, the organization of reliable in vivo investigations with humans requires a sufficient number of volunteers to be included. The obligatory ethical rules are met according to the Declaration of Helsinki (1964) and the subsequent revisions (World Medical Association, 1989) [18,19], which increase confidence in the applied procedures and the obtained data. As a result, the costs of the studies and the characterization of the product quality rise considerably. In contrast, in vitro efficacy tests are among the fastest and cheapest applied approaches. Various model surfaces (polymethyl methacrylate, hydrophobic glass, etc. [18,20]) have been used to characterize the cleansing efficacy of different compositions. New materials have been designed to better mimic the human skin surface [8,21,22].

The surface properties of the substrate to be cleaned have always been treated as the main governing factors for the proper choice of the detergent [20,23,24]. The physicochemical mechanisms of the soil removal from the skin and hair can be found briefly described in review papers and books [23,24,25], with detailed references to the pioneering publications. Varieties of in vitro skin models have been developed and improved in the last 20 years with the aim of decreasing the cost and risk, improving the model relevance, and accelerating the speed of substance testing, especially for topical drug delivery systems [26,27]. The robust skin models have been tested for a variety of cosmetic applications: skin tanning [28]; skin tribology characterization [29]; tactile skin perception [30]; spreading properties of emollients [31]; antiviral activity of organic acids [32], etc. Different cleaning protocols have been applied to model skin surfaces to support patent claims [33]. In the literature, the cleansing efficacy is mostly considered as a macroscopic characteristic, and to our knowledge, there are no discussions on the molecular mechanisms of soil removal and on the impact of the surfactant type on the particular mechanism.

Our investigation aims to demonstrate that model experiments with artificial skin could be applied and used to investigate the physicochemical mechanisms of soil removal, and to discriminate the effects of different types of surfactants in the cleansing compositions. For that purpose, we used the artificial skin Vitroskin^®^ [34], which “has been formulated to have topography, pH, critical surface tension, chemical reactivity, and ionic strength that is similar to human skin”. Although it has been used in many studies, only a single set of data has been found for its particular surface energy value [8]. Recently [35], we determined the surface energy of hydrated and non-hydrated Vitroskin^®^ in order to compare the respective values with the available data for human skin in vivo, and to elucidate the effect of skin hydration. The hydration slightly increased the total surface energy of Vitroskin^®^ (from 35.4 to 37.1 mN/m using the method of Wu [35,36]) and the obtained values are close to the value of 35.8 mN/m reported in [8] and to values for human skin (from 30 mN/m to 40 mN/m [14,17]). Thus, Vitroskin^®^ is a suitable substrate to model the processes of human skin cleansing.

To distinguish the effects of different surfactant types, the following commercial surfactants are used in the present study: anionics, sodium laureth sulfate (SLES), disodium laureth sulfosuccinate (DSLSS), sodium dodecyl sulfate (SDS); cationic, dodecyl trimethyl ammonium bromide (DTAB); and nonionic coco glucoside (CG). The systematic investigations with two soils: sebum (a mixture of hydrocarbons, fatty acids, esters, etc.) and dimethicone (polydimethyl siloxane frequently added to skin and hair conditioning and decorative cosmetics [1,3,31]), demonstrate a clear impact of the compositions on the soil removal mechanisms and efficacy. In addition, the results with sample cleansing formulations manifest the benefits of the model experiments to real product development.

## 2. Results

### 2.1. Surface and Interfacial Tension Isotherms of the Used Surfactants

**Figure 1:**
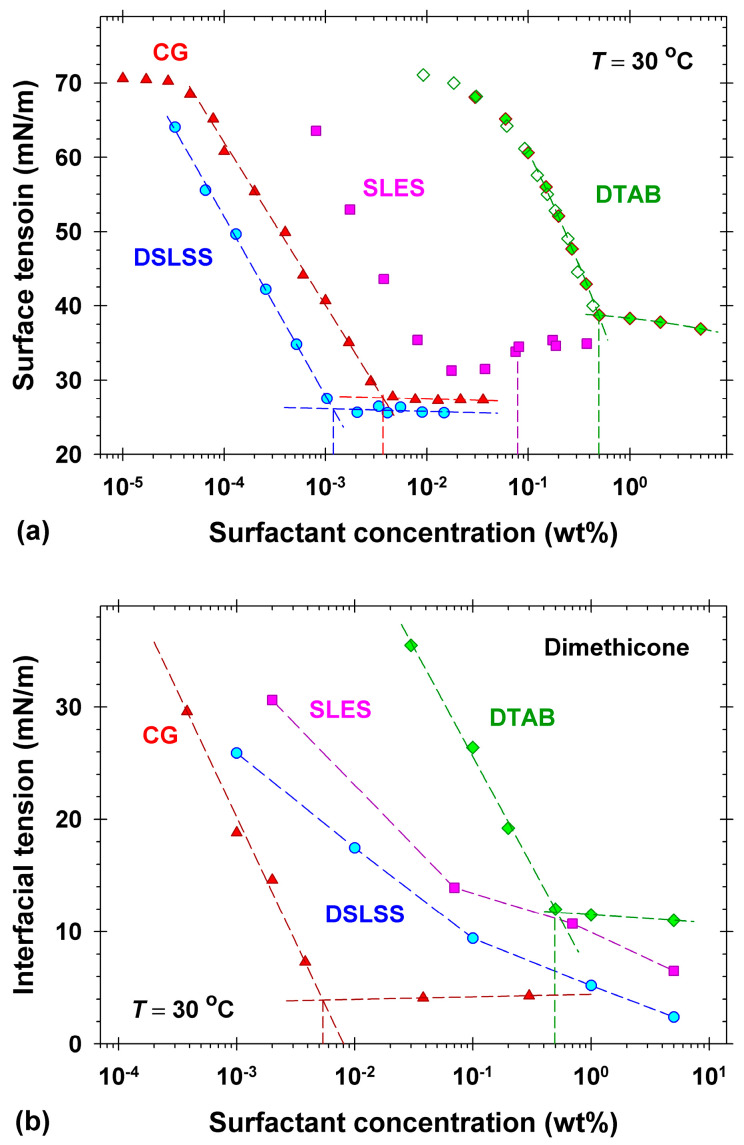
(**a**) Surface tension isotherms of DSLSS (disodium laureth sulfosuccinate), CG (coco glucoside), SLES (sodium laureth sulfate), and DTAB (dodecyl trimethyl ammonium bromide). All measurements were performed at 30 °C by the du Noüy ring method, except for DTAB, where the maximum bubble pressure method was applied. The empty green diamonds show experimental data measured by the du Noüy ring method in Ref. [39]. (**b**) Interfacial tension of selected solutions against dimethicone measured by the pendant drop method at 30 °C.

Surface properties of the surfactants were compared in order to further analyze the impact on their cleansing efficiency. The surface tension isotherms of four of the surfactants used in deionized (DI) water are shown in Figure 1a. Figure 1b represents the interfacial tension isotherms of the same surfactants at the water/dimethicone interface. The measurements were performed at 30 °C, in order to be close to the slightly warm water frequently used for skin cleansing. Weight concentrations are used on the plots since these are mostly used in practical applications and product preparations [3,20,33]. Note that the used concentrations correspond to the active substance in the raw material, which might not be the same as in the industrial recipes [33]. The comparison of the surface tension isotherms (Figure 1a) shows that disodium laureth sulfosuccinate (DSLSS) has the highest surface activity, whereas the cationic surfactant DTAB is the least surface active and has the highest critical micelle concentration (CMC). All CMC values from the surface tension isotherms (indicated by vertical dashed lines in Figure 1a) are listed in Table 1. The values of CMC for DTAB and coco glucoside (CG), determined from the interfacial tension measurements (Figure 1b), are also shown in Table 1, along with the pH range of all solutions (see Figures S1–S4).

The determined CMCs of DTAB from the surface tension, the interfacial tension, and the electrolytic conductivity (Figure S1) data coincide well with the literature data [37,38,39]. The solid symbols for DTAB in Figure 1a are measured at 30 °C by the maximum bubble pressure method (MBPM), and the empty symbols show experimental data measured by the du Noüy ring method in Ref. [39] at 25 °C. The good coincidence of the CMCs suggests that the used sample of DTAB is relatively pure and does not contain additional indifferent electrolytes (salts). DTAB solutions have the highest surface tension and the highest interfacial tension (11 mN/m) with dimethicone above the CMC compared to the other surfactants.

The isotherm of the anionic sodium laureth sulfate (Figure 1a) has a pronounced minimum signifying the presence of nonionic admixtures [40], most probably due to hydrolysis [41,42]. The vertical dashed line in Figure 1a shows the CMC value obtained from the electrolytic conductivity data (Figure S2 and Table 1), which corresponds exactly to the CMC of pure SLES aqueous solutions, CMC = 0.079 wt% [43]. A neutral pH within the range of 6 to 7 is measured for SLES aqueous solutions. Note that the nonionic admixtures in SLES form micelles at 0.02 wt%. Mixed micelles containing both ionic and nonionic components appear for concentrations above 0.02 wt%, in which the molar fraction of the ionic component increases with the rise in concentration up to 0.079 wt% [44]. The oil solubility of the nonionic admixture in SLES affects the interfacial tension (solution/dimethicone), and it decreases monotonically in the entire concentration range studied, reaching 6.5 mN/m at 5 wt%, as shown in Figure 1b. That is why no CMC value, but rather a CMC range for the dimethicone/water interface, is shown in Table 1 for SLES.

The used nonionic CG is a mixture of C8-C16 fatty alcohol glucosides with a low value of the CMC (0.0037 wt%, Figure 1a) determined from the surface tension isotherm. The higher CMC value (0.0054 wt%, Figure 1b) and the low interfacial tension (4.1 mN/m) above the CMC obtained from the interfacial tension isotherm show that, most probably, the admixtures of long-chain alcohols in the used CG sample are dissolved in the dimethicone [45]. The nonionic CG had a significant alkaline pH (Figure S3, Table 1). The high pH of glucoside surfactants typically results from the synthesis process that includes bleaching with either bleach or hydrogen peroxide [46].

The technical grade DSLSS is a mixture of surfactants with C10–C16 hydrocarbon tails and EO groups, which contains admixtures of the nonionic surface active laureth-2, sodium sulfate, and sodium sulfite [47]. The surface tension isotherm of disodium laureth sulfosuccinate (Figure 1a) has no minimum, and the determined CMC (0.0012 wt%) is the lowest compared to the other surfactants. The relatively low values of the surface tension (26 mN/m) above the CMC might be a result of the presence of laureth-2. The very high molar conductivity below the CMC (Figure S4) suggests that the used DSLSS sample contains a high concentration of indifferent electrolytes (sodium sulfate). Note that disodium lauryl sulfosuccinate forms vesicles [48], and the respective surface tension monotonically decreases with the rise in concentration. In contrast, the surface tension isotherm of DSLSS has a well-pronounced kink point, and the CMC value is well determined. The available nonionic surface-active admixtures in the DSLSS sample are partially oil-soluble because the interfacial tension of DSLSS solution/dimethicone monotonically decreases with concentration, reaching the lowest value of interfacial tension (2.4 mN/m at 5 wt%) compared with the other surfactants. Similarly, for SLES, the CMC range for the dimethicone/water interface is shown in Table 1.

### 2.2. Oily Soils Removal from Vitroskin^®^ in Single Surfactant Solutions

**Figure 2:**
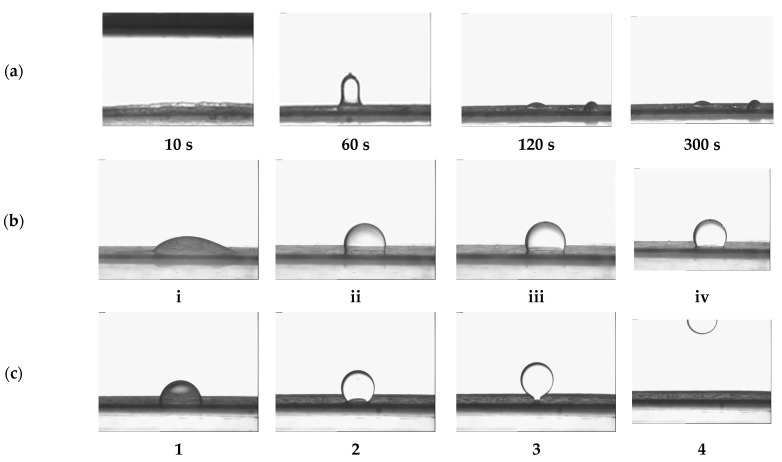
Images of (**a**) sebum soil at different times, as shown below each image, after immersion in a solution of 0.07 wt% SLES. (**b**) Dimethicone droplet after the addition of 1 wt% SLES solution; numbers under each image (**i**–**iv**) correspond to time, as shown on Figure 3. (**c**) Dimethicone droplet after the addition of 1 wt% 1:3 SLES+CG; numbers under each image (**1**–**4**) correspond to time, as shown on Figure 3.

**Figure 3:**
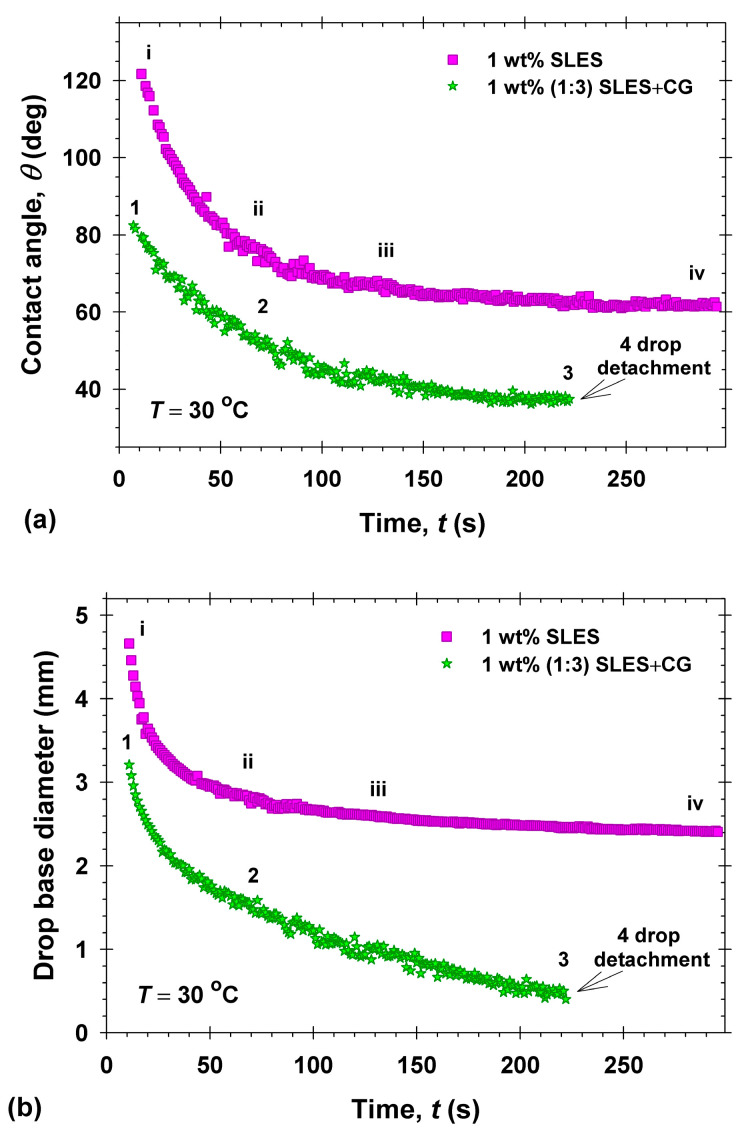
Three-phase contact angle (**a**) and drop base diameter (**b**) vs. time for 1 wt% SLES (no detachment of the oil drop, Figure 2b images corresponding to i, ii, iii, iv) and 1 wt% 1:3 SLES + CG (detachment of the oil drop, Figure 2c images corresponding to 1, 2, 3, 4).

We performed direct observations of soil removal from Vitroskin^®^ with two different soils—model sebum and dimethicone. The following mechanisms of soil removal were observed with the studied soils: emulsification of the sebum after contact with some of the surfactant solutions (Figure 2a), drop shrinking (rolling, Figure 2b,c), and eventual detachment (Figure 2c) in the case of dimethicone. No emulsification of the dimethicone drops on the skin was found, in contrast to the observations with sebum. The contact areas between the dimethicone drops and the substrate decrease slowly over time, *t*, to the equilibrium state (Figure 2b) or to the full drop detachment (Figure 2c), depending on the type of surfactants in the solutions. Figure 3a,b present the changes in the three-phase contact angle through the aqueous phase, *θ*, and the drop base diameter, *d*, with time, *t*, for a dimethicone droplet on artificial skin in a solution of 1 wt% SLES (squares) and 1 wt% (1:3) SLES+CG (stars). In the case of 1 wt% SLES aqueous solution, we did not observe detachments of the droplets from the substrate (cf. Figure 2b). Both the contact angle, *θ*, and the base diameter, *d*, decreased after the addition of the surfactant solution for about 60 s approaching their equilibrium values (*θ* = 61.85 ± 0.35° and *d* = 2.41 ± 0.01 mm determined with double exponential decay fit for the droplet shown on Figure 2b, squares on Figure 3). In the presence of a mixed 1 wt% (1:3) SLES+CG solution (Figure 2c), the three-phase contact angle and the drop base diameter continuously decreased for the initial 120 s from *θ* = 82° and *d* = 3.2 mm to *θ* = 44° and *d* = 1.1 mm (Figure 3). For 120 s < *t* < 230 s, the contact angle slowly dropped to *θ* = 37°, while the contact line shrank rapidly to the moment of the dimethicone drop detachment (*d* = 0.4 mm at *t* = 230 s), see Figure 3 and image number 4 in Figure 2c. This behavior exactly corresponds to the so-called “roll-up mechanism” of soil cleaning [23,25,49,50,51].

The emulsification mechanism [23,24,25] was only observed with the sebum soil (as illustrated in Figure 2a) when the interfacial tension was low enough, as we have shown in our previous study [35]. The fatty acids present in the sebum most probably mix synergistically with some surfactants, forming complex amphiphilic structures and effectively decreasing the interfacial tension. Thus, we have observed emulsification with SLES at relatively low concentrations, while DSLSS has not been capable of emulsifying the sebum even at very high concentrations [35]. Further understanding of the surfactant–soil structuring could be performed with a complex instrumental investigation [52,53].

The roll-up mechanism observed with dimethicone soil allows for the straightforward comparison of surfactant efficiencies [35,49]. For that reason, we performed systematic observations of dimethicone droplets on Vitroskin^®^ in single surfactant solutions varying the respective concentration from at least 10 times below the CMC up to 10× CMC.

The oily soil removal by the rolling-up mechanism is explained by the changes in surface energy and the interfacial tension, leading to simultaneous changes in the three-phase contact angle and the contact area [17,23,49,50]. At equilibrium, the contact angle through the water phase is related to the substrate water, $\gamma_{\mathrm{SW}}$, and the substrate oil, $\gamma_{\mathrm{SO}}$, surface energies and the water–oil, $\gamma_{\mathrm{WO}}$, interfacial tension values by the Young–Laplace equation [17]:

(1) $$\;cos\theta =\left(\gamma_{SO}-\gamma_{SW}\right)/\gamma_{WO}$$

Thus, a plot of cos*θ* versus $\gamma_{WO}$ appropriately illustrates the differences between the used surfactants and the wetting processes [16,35]. For convenience, the full drop detachment is typically denoted as cos*θ* = 1.

**Figure 4:**
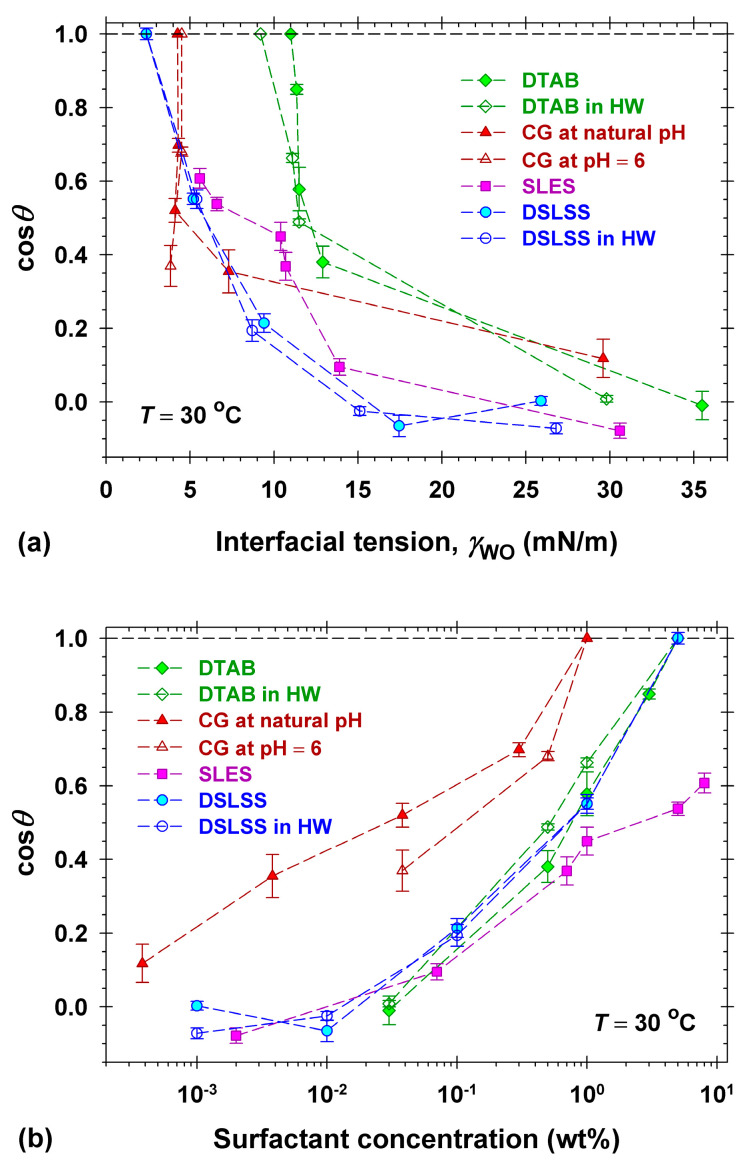
Cosine of the three-phase contact angle through the water phase, *θ*, of dimethicone droplets on Vitroskin^®^ vs. (**a**) the interfacial tension, $\gamma_{\mathrm{WO}}$, and (**b**) the surfactant concentration in the aqueous solutions. A series of solutions was prepared in DI water with the anionic surfactants SLES (squares) and DSLSS (circles), the nonionic surfactant CG (triangles), and the cationic surfactant DTAB (diamonds). The experimental results for two series with DSLSS (empty circles) and with DTAB (empty diamonds) in 150 ppm hard water (HW in the legend) are included. The measurements with CG solutions at fixed pH = 6 (empty triangles) are shown. All measurements were performed at 30 °C.

Figure 4 summarizes the experimental results for the dependence of cos*θ* on the interfacial tension (a) and on the surfactant concentration (b) for deposited dimethicone drops on artificial skin in aqueous surfactant solutions. Two series of measurements were performed with solutions of DTAB and DSLSS in 150 ppm hard water to explore the possible impact of the hardness ions on cleansing efficiency. The natural pH of CG solutions is approximately constant (pH = 10.5–10.8, Figure S3). CG solutions with pH = 6 were also used in order to check the possible effect of pH on the dimethicone drop removal. The values of cos*θ* were taken at 300 s of contact of the dimethicone drop with the respective solution. If the drop’s detachment occurred for *t* < 300 s, then we plotted cos*θ* = 1 in Figure 4. All experiments were conducted under static conditions without cross flows, additional mechanical forces, or agitation.

The obtained dependence of cos*θ* on $\gamma_{\mathrm{WO}}$ (Figure 4a) clearly demonstrates the expected increase in the cosine with the decrease in the interfacial tension. Values of $\gamma_{WO}$ below 5 mN/m were necessary for the complete drop detachment in the solutions of the anionic DSLSS and the nonionic CG solutions, as observed for 5 wt% DSLSS with $\gamma_{\mathrm{WO}}$ = 2.4 mN/m, and for 1 wt% CG with $\gamma_{\mathrm{WO}}$ = 4.4 mN/m. The interfacial tension of all used SLES solutions was larger than 5 mN/m ($\gamma_{\mathrm{WO}}$ = 5.6 mN/m at the highest concentration of 8 wt%, see Figure 1b), and drop detachments were not detected.

The dependence of cos*θ* on the surfactant concentration (Figure 4b) shows that the nonionic CG (either alkaline or at pH = 6) removes the dimethicone droplets from Vitroskin^®^ at the lowest surfactant concentration compared to the ionic surfactants SLES, DSLSS, and DTAB. The best performance of CG is related to its highest interfacial activity, cf. Figure 1b. CG decreases the interfacial tension, $\gamma_{\mathrm{WO}}$, below 5 mN/m at the lowest concentration compared to all other surfactants. The efficacy of DTAB and DSLSS (both in DI and hard water) with respect to concentration is quite similar—the dimethicone droplets fully detach from the artificial skin for 5 wt% aqueous solutions. In the case of SLES, no dimethicone drop detachment was observed under the static experimental conditions, even at the highest experimental concentration of 8 wt% (squares in Figure 4a). SLES is known for its very low Krafft temperature and effectiveness in hard water [1,20,54], which is why its performance in hard water was not studied.

Notably, the drop’s rolling up in DTAB solutions occurred at remarkably high interfacial tension of about 11 mN/m in both hard and DI water. Cationic surfactants and polymers are widely used as skin and hair conditioners [1,2,55] due to their preferential adsorption onto anionic surfaces. Thus, the adsorption of DTAB on the artificial skin is expected to decrease the surface energy $\gamma_{\mathrm{SW}}$, and as a result, the dimethicone droplets detach from the substrate at relatively high values of $\gamma_{\mathrm{WO}}$, see Equation (1). The electrolytic conductivity data (Figure S1) and the surface and interfacial tension isotherms (Figure 1) of DTAB aqueous solutions suggested that the DTAB sample is relatively pure and there are no surface-active admixtures that could be dissolved in the dimethicone. Thus, the surface energy, $\gamma_{SO}$, does not depend on the DTAB concentration. Using Equation (1), we represent the Gibbs adsorption equation in the following form:

(2) $$\;d\left(\gamma_{WO}cos\theta \right)=d\left(\gamma_{SO}-\gamma_{SW}\right)=-d\gamma_{SW}=k_{B}T\Gamma dlnC$$

Here, $k_{B}$ is the Boltzmann constant, and Γ is the surface excess of the surfactant solution/artificial skin boundary.

**Figure 5:**
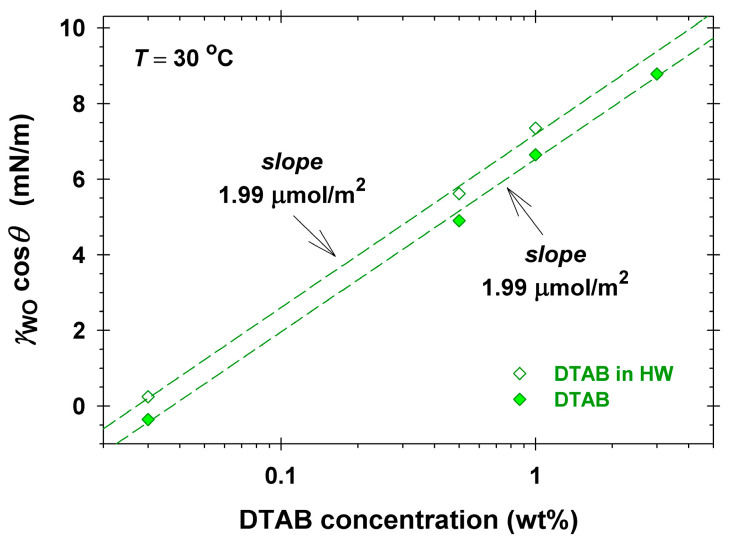
Difference between the surface energies $\gamma_{\mathrm{SO}}$ and $\gamma_{\mathrm{SW}}$ vs. DTAB concentration, *C*, see Equation (2), in DI water and in hard water. The lines represent the linear regression fit through the points. The adsorptions, Γ, determined from the slopes, are shown on the plot.

Figure 5 shows the dependence of the product $\gamma_{\mathrm{WO}}\cos\theta$ on the concentration *C* determined with solutions of DTAB. The dashed lines in Figure 5 correspond to the linear regression according to Equation (2). Both slopes obtained from the data in DI water and hard water are identical: Γ = 1.99 ± 0.05 μmol/m^2^, and the regression coefficients are greater than 0.9995. The obtained surface excess, Γ, is two times lower than the saturation adsorption of DTAB at the solution/air interface [38,39], and it is comparable to values determined on other solid surfaces [56]. This amount is enough to substantially reduce the skin surface energy, $\gamma_{\mathrm{WS}}$, and to allow for drop detachment at high interfacial tension, $\gamma_{\mathrm{WO}}$ (cf. Figure 4a). Note that the dependencies of $\gamma_{\mathrm{WO}}\cos\theta$ vs. surfactant concentration for CG, SLES, and DSLSS (Figure S5) are scattered and cannot be described by Equation (2). Most probably, the presence of dimethicone soluble admixtures in these samples (Figure 1) affects the values of the surface energy, $\gamma_{\mathrm{SO}}$, and the simple form of the Gibbs equation becomes inadequate to describe the respective surface excesses.

In summary, the experimental observations of the behavior of two different soils in different surfactant solutions allowed for the demonstration of two basic mechanisms of soil removal: emulsification and roll-up. The change in the contact angles of the dimethicone drops with the concentration and type of the surfactant outlined the most important factors for the roll-up to occur, namely the interfacial activity of the surfactants. Thus, CG, having the lowest interfacial tension (Figure 1b), has the best performance in removing dimethicone. Other surfactants, such as the used SLES with a low interfacial activity for dimethicone, showed good emulsification of sebum soil, most probably due to synergistic interaction with the fatty acids in the sebum. In practical applications, where different types of soil are met [1,20,23,25], surfactant mixtures are usually set to ensure an optimal cleansing performance.

### 2.3. Dimethicone Soils Removal in Mixed Surfactant Solutions

We prepared mixtures of CG with the three ionic surfactants and performed systematic observations of dimethicone droplets rolling in the mixed surfactant solutions at natural pH to check the impact of the added ionics on the effectiveness of CG solutions.

**Figure 6:**
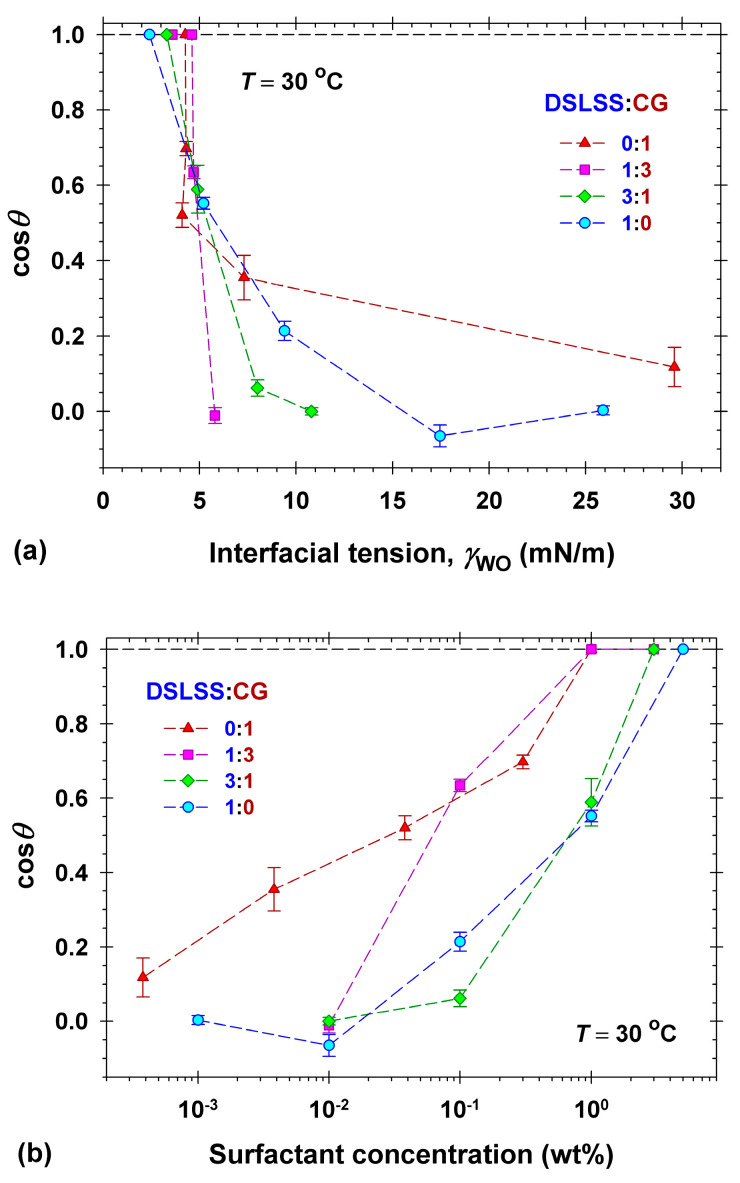
Cosine of the three-phase contact angle through the water phase, cos*θ*, of dimethicone droplets on Vitroskin^®^ vs. (**a**) the interfacial tension, $\gamma_{\mathrm{WO}}$, and (**b**) the total surfactant concentration in mixed solutions of DSLSS and CG at different ratios, as shown in the legend. All measurements were performed at 30 °C.

Figure 6 shows results for the cosine of the aqueous three-phase contact angle with dimethicone droplets on Vitroskin^®^ in DSLSS+CG solutions at anionic/nonionic ratios of 1:3 and 3:1. The obtained data are compared to those with the two surfactants alone, i.e., 1:0 (only DSLSS) and 0:1 (only CG) ratios. The natural pH values of all mixed DSLSS+CG solutions were in the range from 4.8 to 6.2, even at a 1:3 ratio between the two surfactants. The results with solutions of 1:3 and 0:1 DSLSS+CG were almost the same as interfacial tension (Figure 6a) and concentration (Figure 6b) dependencies, except at very low concentrations. One can conclude that the coco glucoside interfacial activity was dominant for the overall removal efficiency. With the 3:1 mixture, 3 wt% total surfactant concentration was necessary to decrease the interfacial tension below 5 mN/m and to roll-up the dimethicone drop. This total surfactant concentration (3 wt%) is lower than the necessary concentration for DSLSS alone (5 wt%) but higher than that for the 1:3 mixture (1 wt%).

**Figure 7:**
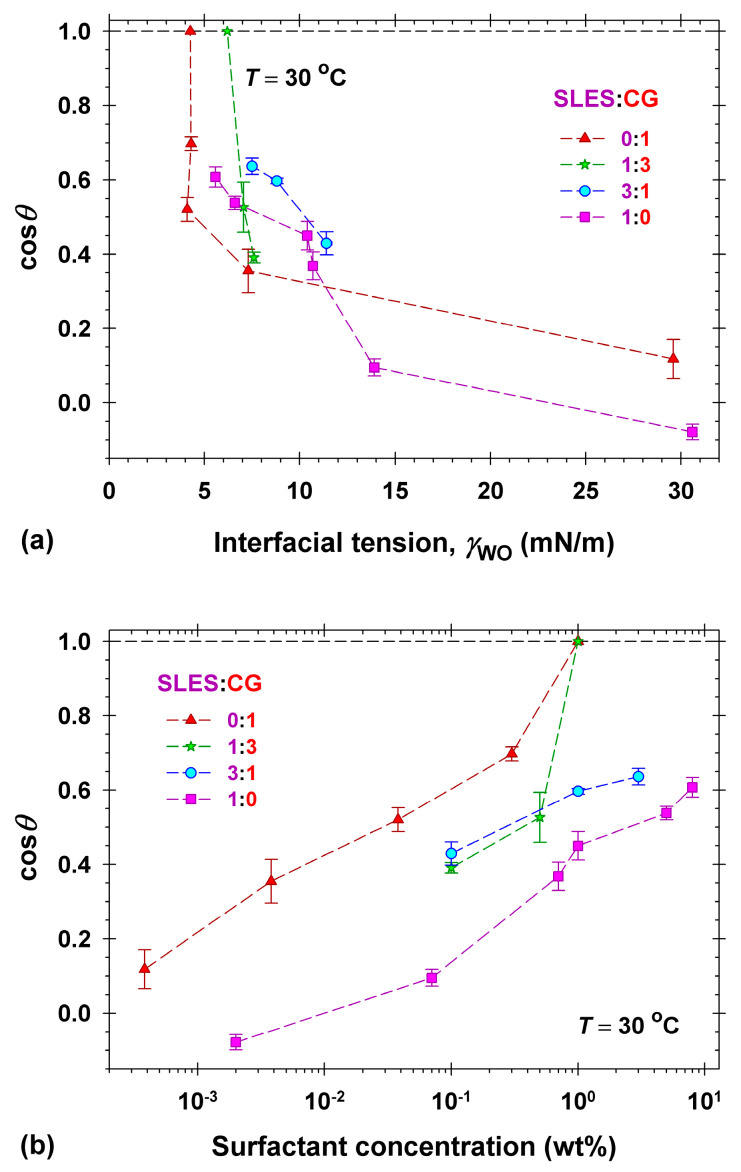
Dependence of the cosine of the three-phase contact angle, cos*θ*, of dimethicone droplets on Vitroskin^®^ on (**a**) the interfacial tension, $\gamma_{\mathrm{WO}}$, and on (**b**) the total surfactant concentration in mixed solutions of SLES and CG. All measurements were performed at 30 °C.

The behavior observed with the mixed solutions of SLES and CG (see the plots in Figure 7) was different compared to that of DSLSS and CG. First, the natural pH values of all mixed solutions were between 10 and 11, even at a 3:1 SLES+CG ratio, and the pHs are controlled by the available CG. Note that the removal efficiency of CG alone is approximately the same at natural pH and at pH = 6, see Figure 4. The mixtures with prevailing CG, i.e., 1:3, successfully detached dimethicone droplets at 1 wt% total surfactant concentration. The mixture with less CG, i.e., 3:1, had lower interfacial tension than SLES solutions (Figure 7a), but the decrease was not sufficient to observe drop roll-up. Note that the interfacial tension value, $\gamma_{\mathrm{WO}}$, is not the only factor to influence the contact angle in this case. The mixed solution of 1:3 SLES:CG achieved complete oil removal at a concentration of 1 wt% and $\gamma_{\mathrm{WO}}$ = 6.3 mN/m, while in 8 wt% SLES solution with $\gamma_{\mathrm{WO}}$ = 5.5 mN/m no oil release was observed. In such case, one could expect change in the surface energy of the substrate, see Equation (1), where mixed adsorption layer is formed. The morphology of such layers depends on both structure and concentration of the surfactants [56] and could be a subject of detailed investigations in future studies.

**Figure 8:**
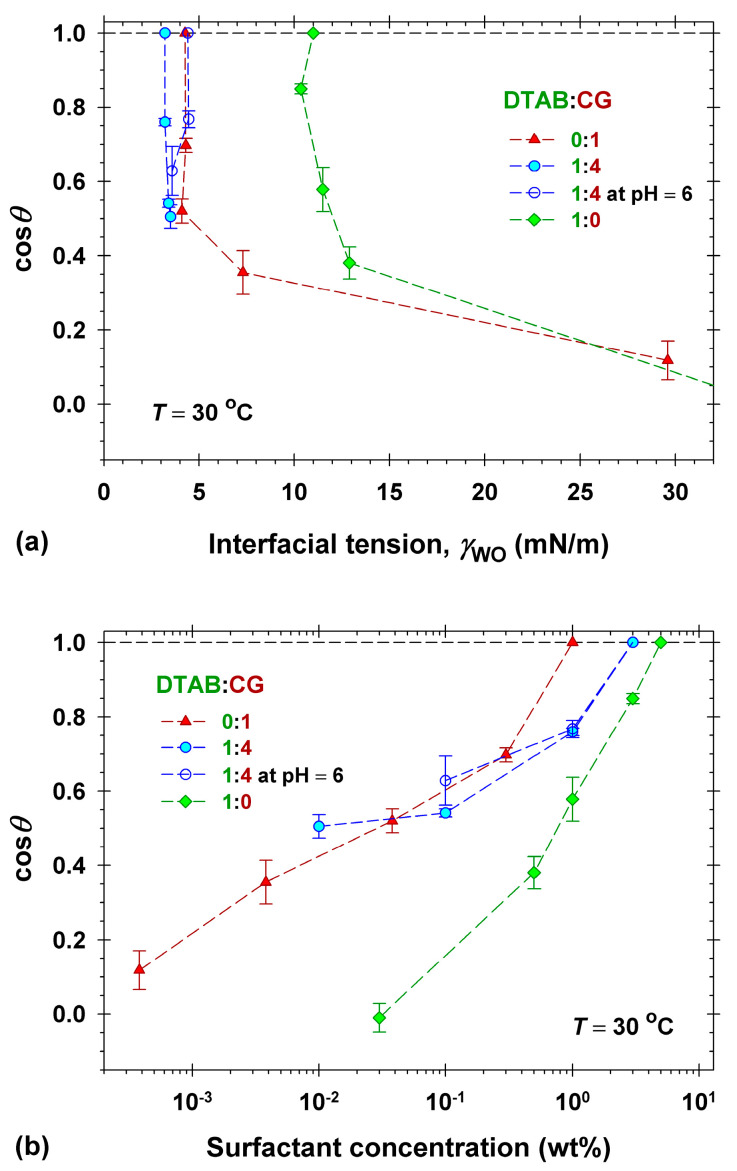
Cosine of the three-phase contact angle through the water phase, cos*θ*, of dimethicone droplets deposited on Vitroskin^®^ vs. (**a**) the interfacial tension, $\gamma_{\mathrm{WO}}$, and (**b**) the total surfactant concentration in mixed solutions of DTAB and CG (*T* = 30 °C).

The effect of the cationic DTAB in the mixed DTAB+CG surfactant solutions on the detachment of dimethicone droplets deposited on Vitroskin^®^ is shown in Figure 8, along with the results obtained for DTAB+CG at a ratio of 1:4. These solutions had an alkaline natural pH at concentrations higher than 0.5 wt% due to the prevailing concentration of CG. The parallel measurements were performed by adjusting the pH of all studied solutions to pH = 6, and the obtained results are included in Figure 8. The lowest value of $\gamma_{\mathrm{WO}}$ of 3.2 mN/m was measured in the alkaline mixed solutions (see Figure 8a); however, the detachment of droplets was observed at 3 wt% total concentration in both alkaline and pH = 6, see Figure 8b.

The mixtures of CG with the anionic surfactants were very effective at an anionic/nonionic ratio of 1:3, and the dimethicone drop detachments occurred at a total concentration of 1 wt% (cf. Figure 6b and Figure 7b). Thus, the synergistic mixing of CG with DSLSS and SLES at a ratio of 1:3 with respect to the dimethicone removal efficiency was observed. In contrast, the addition of DTAB to CG at a 1:4 ratio produced a less effective mixture, leading to drop detachments at a total concentration of 3 wt%. Thus, the antagonistic mixing of CG and DTAB takes place.

**Figure 9:**
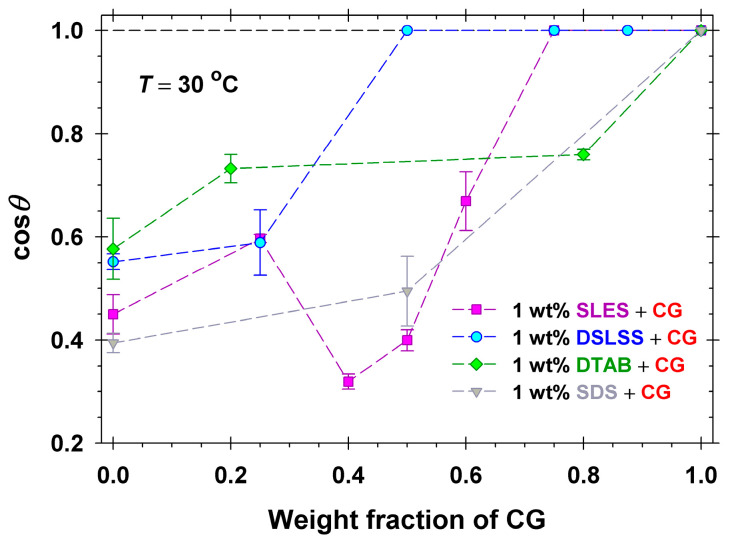
Cosine of the three-phase contact angle through the water phase of dimethicone droplets deposited on Vitroskin^®^ vs. the weight fraction of CG in mixed surfactant solutions (CG with ionic surfactant) at a total concentration of 1 wt% and *T* = 30 °C.

To compare directly the impact of the different ionic surfactants on the effectiveness of CG, we present cos*θ* as a function of the weight fraction of the nonionic CG in the respective mixtures at a total surfactant concentration of 1 wt%; see Figure 9. The addition of all ionic surfactants deteriorates the effectiveness of CG, as seen from the data. No drop detachment was observed in any of the solutions containing the cationic DTAB. Best results were obtained with the mixture of DSLSS and CG, which is effective even at a 1:1 ratio. A peculiar minimum of cos*θ* versus the weight fraction of CG was observed for CG+SLES mixed surfactant solutions. To check whether this minimum was related to the specific interactions of the sulfate head or to the present nonionic admixture (cf. Section 2.1 and Figure 1b), we prepared a mixture with sodium dodecyl sulfate (SDS) at a 1:1 ratio. The results obtained (triangles on Figure 8) were quite comparable to those of the same ratio of SLES and CG. One could summarize that sulfate surfactants SLES and SDS have shown quite different dimethicone soil removal activity: the worst results were obtained at 50 to 60 weight percent sulfate surfactant in the mixtures with CG. The opposite effect has been observed with sebum: SLES showed the best performance by emulsification mechanisms at a relatively low surfactant concentration, most probably acting synergistically with components of the sebum. These results clearly illustrate the importance of the optimization step when formulating cleansing surfactant mixtures for a particular application.

### 2.4. Dimethicone Soils Removal in Model Cleansing Formulations

The cleansing products on the market contain not only surfactants but also humectants to avoid skin dryness, preservatives to ensure consumer safety, and perfume composition (PC) to have a pleasant scent [1,2,3,6,20]. All these additives might interact with the surfactants, leading to product and cleansing deterioration in some cases [3,57].

To demonstrate further the applicability of the proposed procedure to actual product development processes, we performed observations of dimethicone removal from Vitroskin^®^ in simple cleanser formulations. The surfactant base of the formulations was selected using the results for the cleansing performance of the mixed systems presented in Section 2.3. Table 2 presents the compositions of five formulations labeled as F-G (CG containing), F-ES (SLES containing), F-SS (DSLSS containing), F-G+ES (containing a 1:1 mixture of CG and SLES), and F-G+SS (containing a 1:1 mixture of CG and DSLSS). Each formulation contains 12 wt% surfactant, 3 wt% glycerin (humectant), 1 wt% sodium benzoate as a preservative, 0.05 wt% perfume composition, and 1 wt% NaCl. Sodium chloride is a cheap raw material widely used as a rheology modifier for sulfate-containing cleansers [1,3,42,58,59]. We added NaCl with the same concentration to all formulations to ensure relative formulation similarity (no electrolytes in the raw materials have been quantified). The pH of the formulations was adjusted in the range from 5 to 5.5 by means of citric acid. The formulations were prepared one day prior to use. The observations of dimethicone droplets on Vitroskin^®^ were performed after a 12-fold dilution to a total surfactant concentration of 1 wt%. Note that the cleansing formulations with DSLSS (F-SS and F-G+SS) were turbid; however, after dilution, the turbidity was negligible, and optical observations were possible. The solutions of the surfactant DSLSS in water were transparent. The turbidity in the model cleanser formulations most probably resulted from specific interactions of DSLSS either with the preservative Na benzoate or with the used perfume composition.

The measured three-phase contact angles of dimethicone droplets on Vitroskin^®^ in the 12-fold diluted model cleansers are shown in Table 3. For comparison, the contact angles measured with 1 wt% surfactant solutions in DI water are also shown. The determined values of the interfacial tension, $\gamma_{\mathrm{WO}}$, and the pH of the solutions are included in Table 3. The pH slightly changed after the formulations were diluted with water, but the variations were less than 0.5 units, which is not expected to impact the results.

The results in Table 3 show a very good correlation between the observed droplet behavior and the surfactant composition used, except for the mixture of DSLSS and CG (cf. the last two rows in the table). The weaker drop shrinkage, i.e., a higher contact angle, *θ*, in the formulations with DSLSS compared to the aqueous solutions of DSLSS, could be due to the deteriorating effect of the ingredients, seen as turbidity in these formulations.

Overall, the observations of dimethicone droplets on Vitroskin^®^ in the prepared formulations confirmed the excellent drop removal by CG, the surfactant with the best interfacial activity. In addition, the issue of the possible negative impact of some formulation ingredients on the cleansing was well exhibited with the formulations of DSLSS.

## 3. Discussion

The comparison of the cleansing effectiveness of formulations in the model experiments we performed with dimethicone soil on Vitroskin^®^ allowed for good discrimination between their performance: F-G > F-G+SS > F-G+ES ≈ F-SS ≥ F-ES. The obtained rating of the formulation’s performance corresponds qualitatively well to the rating of its interfacial activity (cf. Figure 1b) and is very different from what one could expect based on the surface activity and the CMC values (Figure 1a). The absence of clear impact of the CMC on the surfactant performance is well known in the practice, and the optimization of products is mainly performed by direct examination of the cleaning of colored soils in vivo [18,19] or on selected surfaces [8,20,32,33], e.g., by cloth sweeping or by machine rubbing [18,19,33], and the effectiveness is measured by colorimetry [18,19,20,33]. Although the latter approach looks like a straightforward choice for the industry, it does not allow for the understanding of the intimate mechanisms and the role of the components in the process. The complication with the measurements in vivo represents another challenge, especially when developing makeup removers, for example. Some of the developed artificial skin models are a good alternative to use, as applied widely already [8,32,33]. The model experiments performed by us with Vitroskin^®^ revealed further important details. The dimethicone removing capacity of the cationic surfactant DTAB was found to be comparable to that of the anionic DSLSS and much better than that of SLES (cf. Figure 4a). The determined adsorption of DTAB (1.99 μmol/m^2^, see Figure 5) on the interface of artificial skin/water decreased most significantly the skin surface energy, allowing for dimethicone removal at relatively high interfacial tension (Figure 4b). Good cleaning properties have been documented for other cationics in detergent formulations [60,61]. Cationic surfactants are present in almost all contemporary micellar water products on the market.

Anionic and nonionic surfactants also adsorb on the skin surface; however, the adsorbed amount could not be determined quantitatively, as performed for the cationic DTAB (see Equation (2) and Figure 8), due to the presence of significant oil-soluble fractions that change the surface energy of the oil/skin interface as well. These oil-soluble admixtures influenced the interfacial activity and the overall dimethicone removal efficiency. The surfactant raw materials usually contain admixtures remaining after the synthesis and/or purification step of the substance [42]. These admixtures could significantly change the surface adsorption and cleaning efficacy, as demonstrated in the presented experimental results and analyses.

The interfacial activity and the cleansing depend on the soil phase as well. The anionic SLES decreased the interfacial tension sebum/water to less than 1 mN/m at not very high concentration, which resulted in sebum emulsification [35]. The same surfactant and a similar SDS could not decrease $\gamma_{\mathrm{WO}}$ enough to roll up dimethicone droplets. The other anionic DSLSS, which is frequently used as a substitute for sulfate surfactants in contemporary cleansing products, successfully rolls up dimethicone from the artificial skin but could not remove sebum [35].

The optimization step of the surfactant mixtures is crucial for obtaining a good cleansing performance at a minimum surfactant concentration. Our model experiments showed that the mixture of CG and DSLSS (1:1) at a total concentration of 1 wt% was quite successful in dimethicone removal (Figure 9); however, the present perfume and/or preservative deteriorated the performance of their formulation (see Table 3). The addition of CG to DTAB also did not result in the best cleaning. Earlier investigators (see Ref. [23] and the references cited therein) have shown that when using scrubbing or wiping off, as in the application of the contemporary micellar water, a contact angle through the aqueous phase of less than 90° is already enough to ensure a good cleaning. Further upgrade of the applied in vitro methodology, by combining it with wiping off measurements and with a larger number of soils, could be applied to practical cleansing product optimization, bringing a deeper understanding of the role of the ingredients.

## 4. Materials and Methods

### 4.1. Materials

The following anionic surfactants were used: SLES (sodium laureth sulfate, commercial name Texapon N70, product of BASF, Ludwigshafen, Germany), DSLSS (disodium laureth sulfosuccinate, Texapon SB3KC, BASF, Ludwigshafen, Germany), and SDS (sodium dodecyl sulfate, SigmaAldrich, Darmstadt, Germany). The cationic DTAB (dodecyl trimethyl ammonium bromide, TCI, Zwijndrecht, Belgium) and the nonionic CG (coco glucoside, Plantacare 818 UP, BASF, Ludwigshafen, Germany) surfactants were also used in our studies. The structural formulas of the surfactants are shown in Table S1. Solutions of the surfactants were prepared in deionized (DI) water from an Elix purification system (Millipore, Darmstadt, Germany) and in hard water containing 150 ppm calcium and magnesium salts. Hard water was prepared according to the procedure after the US Environmental Protection Agency [62]. The used CaCl_2_, MgCl_2_, and NaHCO_3_ were products of Sigma Aldrich.

The model soils were sebum and dimethicone (polydimethylsiloxane AK100, Wacker, Munich, Germany). The artificial sebum was prepared with the following composition [63,64]: 15% Squalene, 25% Wax esters, 2% Cholesteryl esters, 42% Triglycerides, 15% Fatty acids, 1% Cholesterol (all products of Sigma). Dimethicone was chosen as a model soil since it is a key component in decorative cosmetics, including products like lipsticks, gloss, etc. [1,2,3]. Structural formulas of the soil ingredients are shown in Table S2.

As an artificial skin, we used VitroSkin^®^ (purchased from IMS, Bunnell, FL, USA). Pieces of size 1 × 2 cm were cut and used either dry or hydrated as advised by the producers [31]: 16 h in a closed chamber above a water–glycerin solution, 52 g glycerin in 298 g water.

For the preparation of the model cleansing formulation, we used glycerin (Valerus, Sofia, Bulgaria) as a humectant, inorganic salt NaCl (Sigma) as a thickener of the formulation, Na Benzoate (Valerus, Sofia, Bulgaria) as a preservative, and a perfume composition (Crisp spring, Luzi fragrance compounds). To adjust the desired pH of the solution, HCl (Sigma Aldrich, Darmstadt, Germany) or citric acid (Valerus, Sofia, Bulgaria) solutions were used.

### 4.2. Methods

#### 4.2.1. Surface and Interfacial Tension Measurements, pH, and Conductivity Determination

Surface tension isotherms for the surfactants used in deionized water at 30 °C were determined using the du Noüy ring method with a K100 tensiometer (Kruess GmbH, Hamburg, Germany), equipped with an automatic dosing unit and a micro dispenser, and the maximum bubble pressure method with a BP100 (Kruess GmbH, Hamburg, Germany). The interfacial tension of the oils in the chosen surfactant solutions was measured using the pendant drop method with the Drop Shape Analyzer DSA100E (Kruess GmbH, Hamburg, Germany) at 30 °C. Note that the used artificial sebum was liquid at 30 °C.

The pH and conductivity of all solutions were measured by Fisherbrand™ accumet™ AB200 benchtop pH/conductivity meter at 30 °C.

#### 4.2.2. Direct Observations of the Soil Removal from Vitroskin^®^

**Figure 10:**
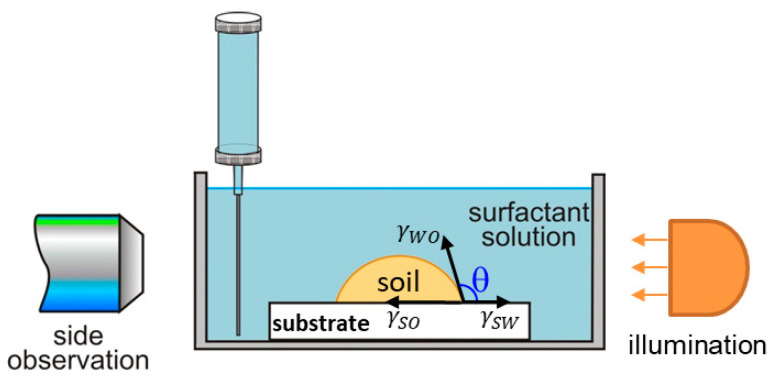
Scheme of experimental setup for direct observations of the soil removal from the surface of Vitroskin^®^. The glass cuvette with the soiled surface and the surfactant solution was mounted on the Drop Shape Analyzer DSA10 (Kruess GmbH, Hamburg, Germany), whose optical system was used for the observations. All experiments were performed at 30 °C.

The observations of soil removal were conducted using the Drop Shape Analyzer DSA10 (Kruess GmbH, Hamburg, Germany) optical system. The methodology and experimental setup (Figure 10) were adapted after Yavrukova et al. [51]. The procedure was as follows: First, Vitroskin^®^ substrate, hydrated in advance, was placed at the bottom of a 50 mL rectangular glass cuvette. Then, a 5 μL soil drop was deposited onto the substrate and allowed to rest for 5 min. Subsequently, 20 mL of surfactant solution at 30 °C was gently added to the cuvette. The shrinking of the contact area between the soil droplet and the substrate (including potential drop detachment) was monitored for 5 min (see Figure 2 and Figure 3). Measurements with each surfactant solution or formulation were conducted at least twice, i.e., on two or more different substrates.

## 5. Conclusions

The performed systematic characterization of soil removal from artificial skin by applying direct optical observations in different surfactant solutions allowed for the demonstration of two basic cleansing mechanisms: emulsification and roll-up. Emulsification occurred in systems with very low (below 1 mN/m) interfacial tension, $\gamma_{\mathrm{WO}}$, as for the sebum and SLES solutions. Dimethicone soil was removed by the roll-up mechanism, and no emulsification was observed.

The effectiveness of the roll-up depends on the interfacial activity of the used surfactants and on their adsorption on the soiled surface. Thus, the cationic DTAB, adsorbing strongly on the artificial skin, was able to roll up dimethicone at $\gamma_{\mathrm{WO}}$ as high as 11 mN/m. The anionic SLES and DSLSS, and the nonionic CG had lower adsorption on the skin than DTAB, but reduced the oil/water interfacial tension more significantly, and successfully rolled up dimethicone at $\gamma_{\mathrm{WO}}$ below 5 mN/m. The used DTAB did not contain oil-soluble components, as evidenced by the conductivity, surface, and interfacial tension measurements, which allowed for the determination of its adsorption of 1.99 μmol/m^2^ on the Vitroskin^®^ surface.

The nonionic CG fully removed the dimethicone from the skin at a lower concentration compared to the ionic surfactants used. The addition of some fraction of CG to the ionic surfactants was used to obtain good cleaning at decreased concentration of the anionics.

The developed in vitro procedure for cleansing efficacy characterization was applied to a series of simple cleansing formulations based on preselected surfactant mixtures and containing also humectant, salt, preservative, and perfume. The comparison of the cleansing effectiveness of formulations allowed for a good discrimination between their performance. Furthermore, their rating corresponded qualitatively well to the performance of the surfactant mixtures and to the rating of their interfacial activity.

Our investigation demonstrated well that model experiments with artificial skin could be applied and used to investigate the physicochemical mechanisms of soil removal, and to discriminate the effects of different types of surfactants in the cleansing compositions. Future applications of the developed approach to practical formulations would speed up the product development process and would allow for a fast evaluation of new raw materials.

## Figures and Tables

**Table 1:** The critical micellar concentration (CMC) determined from the isotherms (Figure 1) at the air/solution and dimethicone/solution interfaces. The last column presents the pH range of the surfactant solutions used.

Surfactant	CMC, wt%		pH Range
	Air/Water	Dimethicone/Water	
CG	0.0037	0.0054	6.7–10.8
DSLSS	0.0012	>0.1	7.0–5.0
SLES	0.079	>0.1	7.0–6.0
DTAB	0.49	0.49	6.5–5.7

**Table 2:** Composition of the model formulations of cleansing products.

	Composition, wt%				
Substances	F-G	F-ES	F-SS	F-G+ES	F-G+SS
water	qs to 100	qs to 100	qs to 100	qs to 100	qs to 100
CG	12	–	–	6	6
SLES	–	12	–	6	–
DSLSS	–	-	12	-	6
Glycerin	3	3	3	3	3
NaCl	1	1	1	1	1
Na Benzoate	1	1	1	1	1
Citric acid	qs	qs	qs	qs	qs
PC	0.05	0.05	0.05	0.05	0.05

**Table 3:** Contact angle, *θ*, interfacial tension, $\gamma_{\mathrm{WO}}$, and pH of aqueous surfactant solutions (no pH was adjusted, except for CG) and diluted formulations (see Table 2) at a total surfactant concentration of 1 wt%. Full drop detachment was observed when cos*θ* = 1.

Surfactants, 1 wt%	Contact Angle, *θ*, deg	cos*θ*	$\gamma_{WO}$, mN/m	pH
CG	0	1	4.5	6.0
F-G	0	1	4.5	5.6
SLES	63.3	0.45	10.4	6.8
F-ES	63.5	0.44	9.3	5.5
DSLSS	56.5	0.55	5.4	4.8
F-SS	61.2	0.48	5.2	4.7
1:1 CG+SLES	66.5	0.40	7.7	10.4
F-G+ES	61.3	0.48	7.3	5.5
1:1 CG+DSLSS	0	1	5.2	6.8
F-G+SS	47.1	0.68	4.3	4.8

## Data Availability

The data from this study are available upon request made to the corresponding author.

## Supplementary Materials

The following supporting information can be downloaded at: https://www.mdpi.com/article/10.3390/molecules30081813/s1. Figure S1. Electrolytic conductivity, *κ*, and pH of DTAB aqueous solutions vs DTAB molar concentration, *C*. Figure S2. Electrolytic conductivity, *κ*, and pH of SLES aqueous solutions vs SLES molar concentration, *C*. Figure S3. Electrolytic conductivity, *κ*, and pH of CG aqueous solutions vs CG molar concentration, *C*. Figure S4. Electrolytic conductivity, *κ*, and pH of DSLSS aqueous solutions vs DSLSS molar concentration, *C*. Figure S5. Dependence of $\gamma_{\mathrm{WO}}\cos\theta$ on the surfactant concentration for CG, SLES, and DSLSS solutions. Table S1. Structural formulas of the used surfactants. Table S2. Structural formulas of the ingredients of the soils.

## References

[1] Baki G., Alexander K.S. (2015). Introduction to Cosmetic Formulation and Technology.

[2] Abe T., Sakamoto K., Lochhead R.Y., Maibach H.I., Yamashita Y. (2017). Psychology of Cosmetic Behavior. Cosmetic Science and Technology: Theoretical Principles and Applications.

[3] Mitsui T. (1997). New Cosmetic Science.

[4] Ananthapadmanabhan K.P. (2019). Amino-Acid Surfactants in Personal Cleansing (Review). Tenside Surf. Det..

[5] Jahan R., Bodratti A.M., Tsianou M., Alexandridis P. (2020). Biosurfactants, Natural Alternatives to Synthetic Surfactants: Physicochemical Properties and Applications. Adv. Colloid Interface Sci..

[6] European Union (2009). Regulation (EC) No 1223/2009 of the European Parliament and of the Council of 30 November 2009 on Cosmetic Products.

[7] Ginn M.E., Noyes C.M., Jungermann E. (1968). The Contact Angle of Water on Viable Human Skin. J. Colloid Interface Sci..

[8] Eudier F., Savary G., Grisel M., Picard C. (2019). Skin Surface Physico-chemistry: Characteristics, Methods of Measurement, Influencing Factors and Future Developments. Adv. Colloid Interface Sci..

[9] Aßmus U., Banowski B., Brock M., Erasmy J., Fitzner A., Kortemeier U., Langer S., Munke S., Schmidt-Lewerkühne H., Segger D. (2013). Impact of Cleansing Products on the Skin Surface pH. IFSCC Mag..

[10] Hawkins S., Dasgupta B.R., Ananthapadmanabhan K.P. (2021). Role of pH in Skin Cleansing. Int. J. Cosmet. Sci..

[11] Mavon A., Zahouani H., Redoules D., Agache P., Gall Y., Humbert P. (1997). Sebum and Stratum Corneum Lipids Increase Human Skin Surface Free Energy as Determined from Contact Angle Measurement: A Study on Two Anatomical Sites. Colloids Surf. B Biointerfaces.

[12] Mavon A., Redoules D., Humbert P., Agache P., Gall Y. (1998). Changes in Sebum Levels and Skin Surface Free Energy Components Following Skin Surface Washing. Colloids Surf. B Biointerfaces.

[13] German G.K., Pashkovski E., Dufresne E.R. (2013). Surfactant Treatments Influence Drying Mechanics in Human Stratum Corneum. J. Biomech..

[14] Elkhyat A., Agache P., Zahouani H., Humbert P. (2001). A New Method to Measure *In Vivo* Human Skin Hydrophobia. Int. J. Cosmet. Sci..

[15] Elkhyat A., Mavon A., Leduc M., Agache P., Humbert P. (1996). Skin Critical Surface Tension: A Way to Assess the Skin Wettability Quantitatively. Skin Res Technol..

[16] Krawczyk J. (2015). Surface Free Energy of the Human Skin and its Critical Surface Tension of Wetting in the Skin/Surfactant Aqueous Solution/Air System. Skin Res. Technol..

[17] Tsujii K., Sakamoto K., Lochhead R.Y., Maibach H.I., Yamashita Y. (2017). Wetting and Surface Characterization. Cosmetic Science and Technology: Theoretical Principles and Applications.

[18] Elsner P., Seyfarth F., Sonsmann F., John S.-M., Diepgen T., Schliemann S. (2014). Development of a Standardized Procedure for Testing the Efficacy of Workplace Cleansers. Contact Dermat..

[19] Sonsmann F.K., Strunk M., Gediga K., Schliemann S., Seyfarth F., Elsner P., Diepgen T.L., Kutz G., John S.M. (2014). Standardization of Skin Cleansing *In Vivo*: Part II. Validation of a Newly Developed Automated Cleansing Device (ACiD). Skin Res. Technol..

[20] Lai K.-Y. (2005). Liquid Detergents.

[21] Ali A., Ringstad L., Skedung L., Falkman P., Wahlgren M., Engblom J. (2022). Tactile Friction of Topical Creams and Emulsions: Friction Measurements on Excised Skin and VitroSkin^®^ using ForceBoard™. Int. J. Pharm..

[22] Lir I., Haber M., Dodiuk H. (2007). Skin Surface Model Material as a Substrate for Adhesion-to-Skin Testing. J. Adhes. Sci. Technol..

[23] Rhein L., Johansson I., Somasundaran P. (2007). Surfactant Action on Skin and Hair: Cleansing and Skin Reactivity Mechanisms. Handbook for Cleaning/Decontamination of Surfaces.

[24] Cornwell P.A. (2018). A Review of Shampoo Surfactant Technology: Consumer Benefits, Raw Materials and Recent Developments. Int. J. Cosmet. Sci..

[25] Lochhead R.Y., Sakamoto K., Lochhead R.Y., Maibach H.I., Yamashita Y. (2017). Basic Physical Sciences for the Formulation of Cosmetic Products. Cosmetic Science and Technology: Theoretical Principles and Applications.

[26] Flaten G.E., Palac Z., Engesland A., Filipović-Grĉić J., Vanić Z., Škalko-Basnet N. (2015). In Vitro Skin Models as a Tool in Optimization of Drug Formulation. Eur. J. Pharm. Sci..

[27] Schmidt F.F., Nowakowski S., Kluger P.J. (2020). Improvement of a Three-Layered In Vitro Skin Model for Topical Application of Irritating Substances. Front. Bioeng. Biotechnol..

[28] Jermann R., Toumiat M., Imfeld D. (2002). Development of an in Vitro Efficacy Test for Self-Tanning Formulations. Int. J. Cosm. Sci..

[29] Bhushan B. (2012). Nanotribological and Nanomechanical Properties of Skin with and without Cream Treatment using Atomic Force Microscopy and Nanoindentation. J. Colloid Interface Sci..

[30] Tang W., Zhang J., Chen S., Chen N., Zhu H., Ge S., Zhang S. (2015). Tactile Perception of Skin and Skin Cream. Tribol. Lett..

[31] Douguet M., Picard C., Savary G., Merlaud F., Loubat-bouleuc N., Grisel M. (2017). Spreading Properties of Cosmetic Emollients: Use of Synthetic Skin Surface to Elucidate Structural Effect. Colloids Surf. B Biointerfaces.

[32] Turner R.B., Biedermann K.A., Morgan J.M., Keswick B., Ertel K.D., Barker M.F. (2004). Efficacy of Organic Acids in Hand Cleansers for Prevention of Rhinovirus Infections. Antimicrob. Agents Chemother..

[33] DeMarco G.M., Ekman-Gunn E., Poccia J.F. (2016). Gel Wipe Composition Comprising a Superabsorbent Gel Fiber. U.S. Patent.

[34] VITRO-SKIN® The Global Standard for Rapid, Predictive In Vitro Testing. https://ims-usa.com/vitro-skin-substrates/vitro-skin/.

[35] Marinova K., Slavova T., Stanimirova R., Danov K. Artificial Skin Characterization for Cleansing Observation and Quantification. Proceedings of the 33rd IFSCC Congress.

[36] Wu S. (1971). Calculation of Interfacial Tensions in Polymer Systems. J. Polym. Sci..

[37] Moulik S.P., Haque M.d.E., Jana P.K., Das A.R. (1996). Micellar Properties of Cationic Surfactants in Pure and Mixed State. J. Phys. Chem..

[38] Christov N.C., Danov K.D., Kralchevsky P.A., Ananthapadmanabhan K.P., Lips A. (2006). Maximum Bubble Pressure Method:  Universal Surface Age and Transport Mechanisms in Surfactant Solutions. Langmuir.

[39] Battal T., Shearman G.C., Valkovska D., Bain C.D. (2003). Determination of the Dynamic Surface Excess of a Homologous Series of Cationic Surfactants by Ellipsometry. Langmuir.

[40] Ranieri D., Preisig N., Stubenrauch C. (2018). On the Influence of Intersurfactant H-Bonds on Foam Stability: A Study with Technical Grade Surfactants. Tenside Surfact. Det..

[41] Lunkenheimer K., Czichocki G. (1993). On the Stability of Aqueous Sodium Dodecyl Sulfate Solutions. J. Colloid Interface Sci..

[42] O’Lenick A.J. (2014). Surfactants: Strategic Personal Care Ingredients.

[43] Vollhardt D., Czichocki G., Rubert R. (1998). Effect of the Molecular Structure on the Adsorption of Alkyl Ether Sulphates and Alkane Ether Sulphonates at the Air–Water Interface. Colloids Surf. A.

[44] Danov K.D., Kralchevsky P.A., Ananthapadmanabhan K.P. (2014). Micelle–Monomer Equilibria in Solutions of Ionic Surfactants and in Ionic–Nonionic Mixtures: A Generalized Phase Separation model. Adv. Colloid Interface Sci..

[45] Catanoiu G., Carey E., Patil S.R., Engelskirchen S., Stubenrauch C. (2011). Partition Coefficients of Nonionic Surfactants in Water/n-Alkane Systems. J. Colloid Interface Sci..

[46] Varvil J., McCurry P., Pickens C., Zoller U., Sosis C. (2009). Production of Alkyl Glucosides. Handbook of detergents. Part F: Production.

[47] Johnson W., Heldreth B., Bergfeld W.F., Belsito D.V., Hill R.A., Klaassen C.D., Liebler D.C., Marks J.G., Shank R.C. (2015). Safety Assessment of Alkyl PEG Sulfosuccinates as Used in Cosmetics. Int. J. Toxicol..

[48] Matsuoka K., Noshiro N., Kuroki H., Tsuyuzaki K., Hashimoto G. (2022). Vesicle Formation of Disodium Lauryl Sulfosuccinate. J. Mol. Liq..

[49] Dillan K.W., Goddard E.D., McKenzie D.A. (1979). Oily Soil Removal from a Polyester Substrate by Aqueous Nonionic Surfactant Systems. J. Am. Oil Chem. Soc..

[50] Kolev V.L., Kochijashky I.I., Danov K.D., Kralchevsky P.A., Broze G., Mehreteab A. (2003). Spontaneous Detachment of Oil Drops from Solid Substrates: Governing Factors. J. Colloid Interface Sci..

[51] Yavrukova V.I., Shandurkov D.N., Marinova K.G., Kralchevsky P.A., Ung Y.W., Petkov J.T. (2020). Cleaning Ability of Mixed Solutions of Sulfonated Fatty Acid Methyl Esters. J. Surfact. Deterg..

[52] Miller C.A., Raney K.H. (1993). Solubilization-Emulsification Mechanisms of Detergency. Colloids Surf. A.

[53] Raney K.H., Benton W.J., Miller C.A. (1987). Optimum Detergency Conditions with Nonionic Surfactants. I. Ternary Water-Surfactant-Hydrocarbon Systems. J. Colloid Interface Sci..

[54] Lim Y.S., Stanimirova R.D., Xu H., Petkov J. (2016). Sulphonated Methyl Ester a Promising Surfactant for Detergency in Hard Water Conditions. HPC Today.

[55] Fernández-Peña L., Guzmán E. (2020). Physicochemical Aspects of the Performance of Hair-Conditioning Formulations. Cosmetics.

[56] Zhang R., Somasundaran P. (2006). Advances in Adsorption of Surfactants and their Mixtures at Solid/Solution Interfaces. Adv. Colloid Interface Sci..

[57] Halla N., Fernandes I.P., Heleno S.A., Costa P., Boucherit-Otmani Z., Boucherit K., Rodrigues A.E., Ferreira I.C.F.R., Barreiro M.F. (2018). Cosmetics Preservation: A Review on Present Strategies. Molecules.

[58] Yavrukova V.I., Radulova G.M., Danov K.D., Kralchevsky P.A., Xu H., Ung Y.W., Petkov J.T. (2020). Rheology of Mixed Solutions of Sulfonated Methyl Esters and Betaine in Relation to the Growth of Giant Micelles and Shampoo Applications. Adv. Colloid Interface Sci..

[59] Mitrinova Z., Alexandrov H., Denkov N., Tcholakova S. (2022). Effect of Counter-Ion on Rheological Properties of Mixed Surfactant Solutions. Colloids Surf. A.

[60] Yavrukova V., Cooban E., Blanco I., Pambou E., Marinova K., Petkov J. (2025). Investigation of the detergency properties of mixtures of biocides and nonionic surfactants using a new simplified hard surface cleaning method. J. Surf. Det..

[61] Brycki B.E., Kowalczyk I.H., Szulc A.M., Brycka J.A. (2018). Quaternary alkylammonium salts as cleaning and disinfectant agents. Tenside Surfactant Deterg..

[62] (2019). Standard Operating Procedure for Preparation of Hard Water and Other Diluents for Antimicrobial Products.

[63] Lu G.W., Valiveti S., Spence J., Zhuang C., Robosky L., Wade K., Love A., Hu L.-Y., Pole D., Mollan M. (2009). Comparison of Artificial Sebum with Human and Hamster Sebum Samples. Int. J. Pharm..

[64] Walters K.A., Roberts M.S., Walters K.A. (2002). The Structure and Function of Skin. Dermatological and Transdermal Formulations.

