# Supplementary material for: Cleansing Mechanisms and Efficacy on Artificial Skin
Source: Molecules. 2025 Apr 17;30(8):1813. doi: 10.3390/molecules30081813 (PMC12029698; doi:10.3390/molecules30081813)
Supplement: Supplementary file 1 [file molecules-30-01813-s001.zip › molecules-3515268-supplementary.pdf]

Supplementary Materials

for the article

Cleansing Mechanisms and Efficacy on Artificial Skin

Authors: Tatiana Slavova, Rumyana Stanimirova, Krastanka Marinova, Krassimir Danov

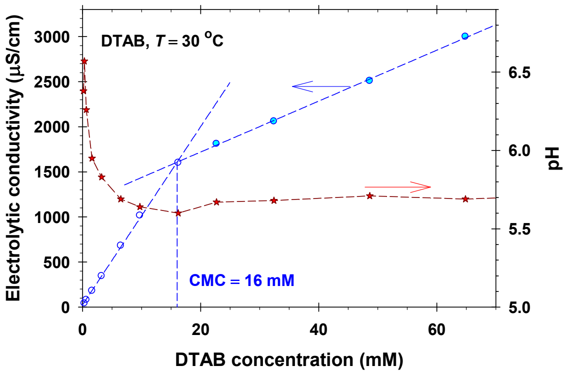

Figure S1. Electrolytic conductivity, κ, and pH of DTAB aqueous solutions vs DTAB molar concentration, C.

The experimental data for the electrolytic conductivity, κ, and pH of DTAB aqueous solutions measured at different DTAB concentrations, C, are summarized in Figure S1. The electrolytic conductivity is a linear function of the concentration below and above the critical micelle concentration (CMC) with the following parameters: slope 98±1 S·cm^2/mol and regression coefficient 0.9995 below the CMC; slope 28±0.6 S·cm^2/mol and regression coefficient 0.9995 above the CMC. The molar conductivity (98 S·cm^2/mol) shows that the solutions do not contain additional indifferent electrolytes (salts).

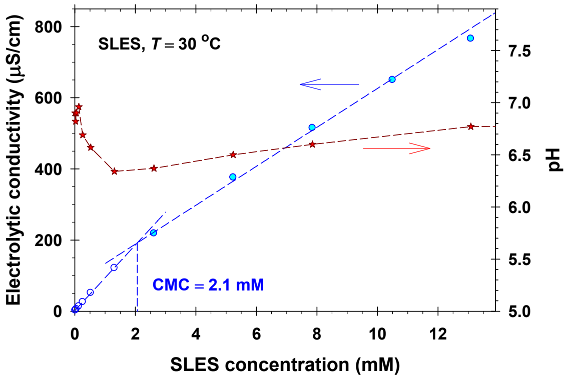

Figure S2. Electrolytic conductivity, κ, and pH of SLES aqueous solutions vs SLES molar concentration, C.

The experimental data for the electrolytic conductivity, κ, and pH of SLES aqueous solutions vs SLES concentrations, C, are shown in Figure S2. The respective parameters of the

linear regressions, κ vs C, below and above the critical micelle concentration (CMC) are: slope 92±0.9 S·cm^2/mol and regression coefficient 0.9998 below the CMC; slope 55±1 S·cm^2/mol and regression coefficient 0.9990 above the CMC. The molar conductivity (92 S·cm^2/mol) is slightly above the expected value for SLES (85 S·cm^2/mol). Thus, the solutions contain negligible concentration of salts, which do not affect the micellization properties of SLES.

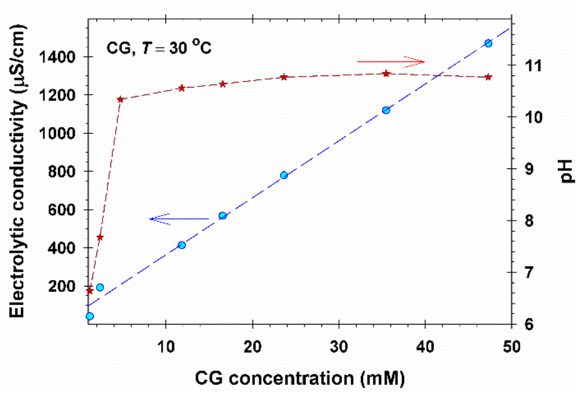

Figure S3. Electrolytic conductivity, κ, and pH of CG aqueous solutions vs CG molar concentration, C.

The experimental data for the electrolytic conductivity, κ, and pH of coco glucoside (CG) aqueous solutions measured at different CG concentrations, C, are summarized in Figure S3. The electrolytic conductivity at approximately equal values of pH is a linear function of the concentration with the following parameters: slope 29.8±0.9 S·cm^2/mol and regression coefficient 0.9955. If one assumes that the CG samples contain NaCl, then the molar ratio of NaCl and CG concentrations should be 0.21. If CG samples contain divalent salts, then the molar ratio between the salt and CG concentrations should be considerably lower. So low concentrations of salts do not affect the micellization properties of CG solutions.

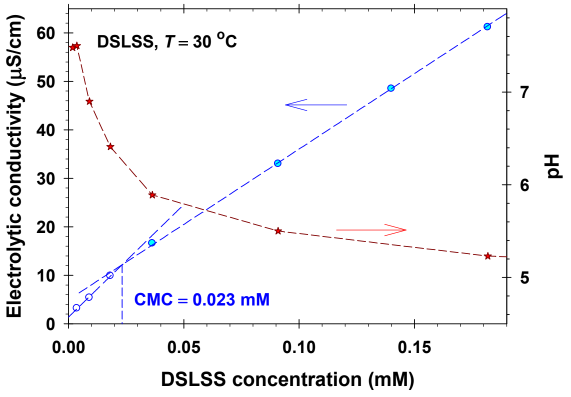

Figure S4. Electrolytic conductivity, κ, and pH of DSLSS aqueous solutions vs DSLSS molar concentration, C.

The experimental data for the electrolytic conductivity, κ, and pH of disodium laureth sulfosuccinate (DSLSS) aqueous solutions vs DSLSS concentrations, C, are shown in Figure S4. The respective parameters of the linear regressions, κ vs C, below and above the critical micelle concentration (CMC) are: slope 464±10 S·cm^2/mol and regression coefficient 0.9990 below the CMC; slope 311±1 S·cm^2/mol and regression coefficient 0.9999 above the CMC. The molar conductivity of DSLSS is expected to be 170 S·cm^2/mol. The rest, 464 – 170 = 294 S·cm^2/mol, corresponds to the presence of divalent indifferent salts. The large values of the molar conductivity above the CMC (311 S·cm^2/mol) also confirm this conclusion. The salt concentration is not negligible and can affect the micellization properties of DSLSS.

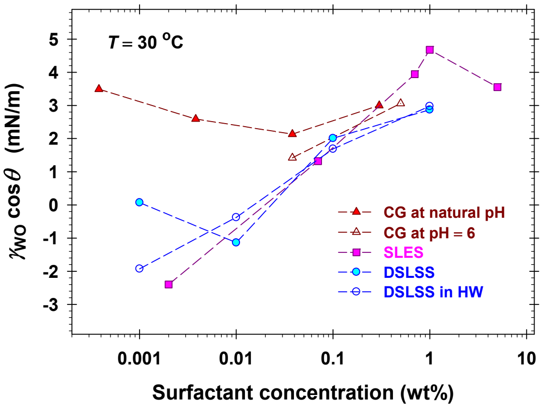

Figure S5. Dependence of $\gamma_{\mathrm{WO}}\cos\theta$ on the surfactant concentration for CG, SLES, and DSLSS solutions.

Table S1. Structural formulas of the used surfactants.

Surfactant	Chemical structure
Coco Glucoside (CG)	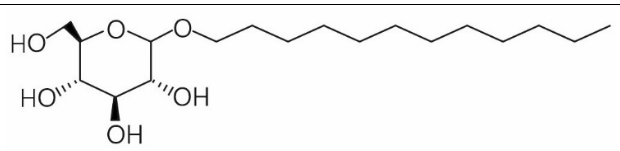
Sodium dodecyl sulfate (SDS)	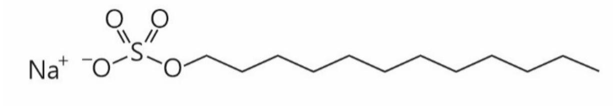
Sodium laureth sulfate (SLES)	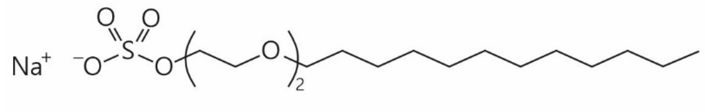
Disodium laureth sulfosuccinate (DSLSS)	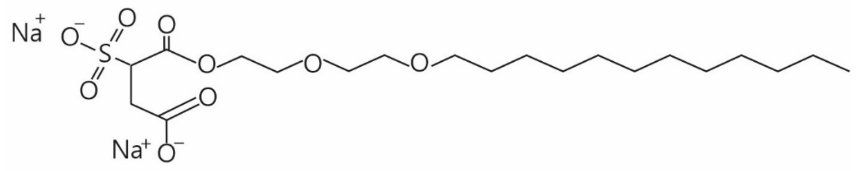
Dodecyl trimethyl ammonium bromide (DTAB)	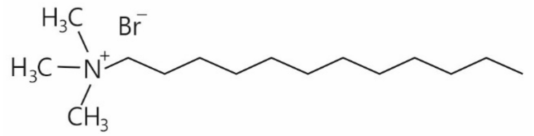

Table S2. Structural formulas of the ingredients of the soils.

Dimethicone	
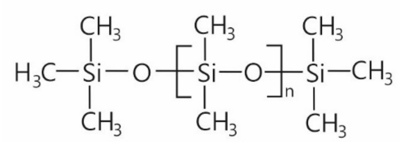	
Sebum ingredients (%)	
Triglycerides (42%)	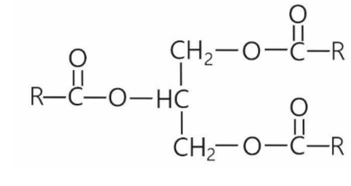
Wax esters (25%)	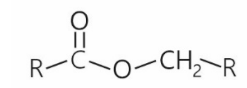
Squalene (15%)	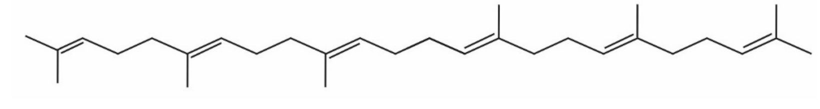

Fatty acids (15%)	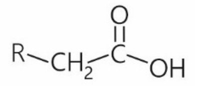
Cholesteryl ester (2%)	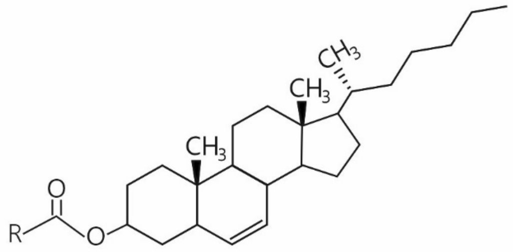
Cholesterol (1%)	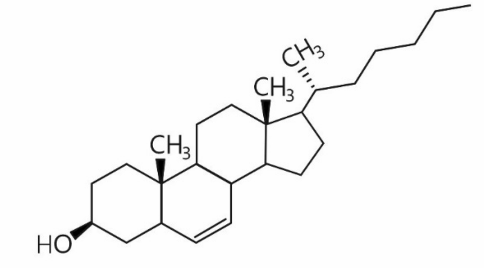
